# Modeling COVID-19 effects on SDGs using system dynamics in Egypt

**DOI:** 10.1007/s11356-022-20019-1

**Published:** 2022-04-05

**Authors:** Mohamed Marzouk, Shimaa Azab, Nehal Elshaboury, Alaa Megahed, Mahmoud Metawie, Mostafa El Hawary, Doaa Ghaith, AbdElMoniem Bayoumi

**Affiliations:** 1 Structural Engineering Department, Faculty of Engineering, Cairo University, Giza, Egypt; 2 Environmental Planning and Development Center, Institute of National Planning (INP), Cairo, Egypt; 3 Construction and Project Management Research Institute, Housing and Building National Research Center, Giza, Egypt; 4 Integrated Engineering Design Management Program, Faculty of Engineering, Cairo University, Giza, Egypt; 5 NAMAA Consult, Road Asset Management Systems Expert, Giza, Egypt; 6 Department of Clinical and Chemical Pathology, Faculty of Medicine, Cairo University, Cairo, Egypt; 7 Department of Computer Engineering, Faculty of Engineering, Cairo University, Giza, Egypt

**Keywords:** COVID-19, Sustainable Development Goals, System dynamics, Impact assessment

## Abstract

The coronavirus disease 2019 (COVID-19) poses a significant threat to achieving the Sustainable Development Goals (SDGs). To address this challenge, a thorough examination of the pandemic’s influence on four SDGs in Egypt is presented in a system dynamic model. The addressed goals are related to no poverty (SDG 1), zero hunger (SDG 2), decent work and economic growth (SDG 8), and climate action (SDG 13). The model is simulated over 35 years extending from 2015 to 2050. Furthermore, a web-based interactive learning environment is developed to analyze the interdependencies among public health activities and study the impacts of possible intervention countermeasures or prevention policies. Indicators including poverty line, food insecurity, gross domestic product (GDP) growth rate, and greenhouse gas (GHG) emissions are evaluated to track Egypt’s performance in relation to SDGs 1, 2, 8, and 13. According to the simulation model, the poverty line will continue to decline until it reaches around 16% by 2050. According to the significant governmental efforts to follow its vision of 2030, Egypt can achieve a decreasing percentage of food insecurity, reaching 3% in 2030, and this percentage will continue to decrease until it reaches full sufficiency by 2050. The GDP growth rate will rise every year until it reaches 13.71% in 2050. With respect to climate, GHG emissions are predicted to fall to roughly 97 Mt CO2-equivalents by 2050. This approach revitalizes debates about the achievement of SDGs amid the crisis and acts as a powerful tool that aids decision-makers in identifying leverage points to avoid the long-term negative repercussions of the crisis on the economy, people, and environment.

## Introduction

The coronavirus disease 2019 (COVID-19) is characterized by its high infection and death potential. As a result, this pandemic has imposed severe dangers to human health, society, and the long-term viability of cities (Ikram et al. 2020; Elavarasan et al. 2020). Since the first case was announced on 30 December 2019, the number of confirmed cases has increased at an alarming exponential pace throughout the globe (Huang et al. 2020). As a result, the World Health Organization (WHO) has declared the newly discovered infectious coronavirus (SARS-CoV-2) a global public health emergency (Li et al. 2020). COVID-19 confirmed cases had surpassed 200 million by the start of September 2021, with over 4 million deaths globally (Worldometer 2021a). As of 8 October 2021, Egypt had the fourth-highest number of confirmed cases in Africa, with 309,934 confirmed cases (Worldometer 2021b). This sparked enormous pressure on governments to find strategies to effectively prevent and restrict the virus spread and the negative associated consequences (World Health Organization, 2020a).

The United Nations (UN) announced the Sustainable Development Goals (SDGs) to be met over a 15-year period, beginning January 1, 2016, and ending December 31, 2030. The 2030 Agenda is a set of 17 SDGs with 169 objectives aimed at making the world more sustainable, resilient, and prosperous (Mouneer 2021). The COVID-19 jeopardizes the growth prospects of developing countries to achieving the SDGs by 2030 (UN Department of Economic and Social Affairs 2020). This problem can be seen as a major setback for the core goals of sustainable development, which are inclusiveness and leaving no one behind (Runde et al. 2020). It is vital to re-establish global unity and common commitment in order to revive momentum toward the 2030 Agenda for Sustainable Development (Barbier and Burgess 2020). In this regard, the UN has established a strategy in an attempt to combat the epidemic. The plan calls on the world’s most powerful countries to take bold legislative steps and provide technical and financial help to the world’s poorest and most vulnerable citizens (UN News 2020).

The COVID-19 epidemic has proven to be associated with ramifications ranging from public health to economy. According to the “Sustainable Development Goals Report 2020,” a pandemic outbreak in 2020 would push 71 million people back into poverty, rising the worldwide poverty rate for the first time since 1998. Healthcare disruptions and restricted access to food and nutrition services have put the refugees, immigrants, aged, and handicapped in vulnerable health and socioeconomic situations. The number of children under the age of 5 and maternal deaths was expected to rise in 2020. The main findings imply that effective institutional governance aids in the eradication of poverty and economic inequality in society (Coccia 2021a). Accordingly, different isolation measures have been implemented by countries all around the world (Chaudhry et al. 2020). These measures have wreaked havoc on the global economy and trade. There was a recovery in international commerce due to the lifting of the embargo and the acceleration of economic activity. However, experts have warned that any economic recovery might be hampered by the ongoing epidemic (World Health Organization 2020b).

This virus has a debilitating effect on the supply chain sustainability, sustainable human resource management, tourism investment and consumption, and economy’s productive capacity (Bai and Ran 2022; Liang et al. 2022). Human, capital, and natural resources are necessary for the production of products. Therefore, a drop in the productivity of one area certainly hinders the achievement of the other sector’s objectives. Furthermore, the prevention measures such as school closures and laws that encourage workers to work remotely might increase worker absenteeism (Keogh-Brown 2014). However, no rigorous study of the performance of online work platforms in sustaining productivity had been conducted. Based on the functioning of global supply chains, online platforms will be unable to maintain the amount of economic activity before the epidemic (Ivanov 2020). Furthermore, the lockdowns and quarantine measures, which were imposed from the outbreak start until the virus stabilizes, would increase gross domestic product (GDP) loss. Examples of these measures are avoiding public transportation and international travel, avoiding entertainment events, and limiting shopping to essentials (Chakraborty and Maity 2020). Accordingly, it is essential to reduce the negative economic effect of pandemic outbreak reactions despite the paramount importance of public health (Xiao and Torok 2020). Furthermore, pursuing green public procurement not only encourages economic growth but also benefits the environment (Nundy et al. 2021).

The COVID-19 pandemic has underlined the need of implementing the 2030 Agenda for Sustainable Development (Coccia 2021b). GDP per capita, healthcare spending, and air pollution level are all important determinants in the COVID-19 mortality rate (Coccia 2021c). The actions that guided the achievement of the SDGs are critical to a faster recovery from the COVID-19 epidemic. The goal of sustainable development is to balance the social, economic, and environmental objectives (Clark and Dickson 2003; Elliott 2012). In this regard, the 17 SDGs were created to develop a unified path that contributes to the current and future wellbeing of the population (Sachs 2012; Desa 2016). All African nations were considered to be lagging on SDG objectives, and this epidemic will reverse the achieved progress (Ekwebelem et al. 2021). The Egyptian government has taken several actions to reduce the impact of the COVID-19 epidemic on human health. Establishing a curfew, social distance, delegating isolation sections in health institutions, and permitting work/study from home are examples of these strategies (Marzouk et al. 2021). As a result, COVID-19 has slowed Egypt’s progress toward achieving the SDGs (Ministry of Planning and Economic Development 2021).

Some research efforts were exerted to study the impact of COVID-19 on SDGs. Elavarasan et al. (2022) proposed a hybrid qualitative and quantitative framework to assess the impact of COVID-19 on SDGs by examining its effect in every goal. The quantification was performed in terms of the targets of the SDGs with the aid of ranking methodology. Ranjbari et al. (2021) suggested five research directions for sustainable development corresponding to the SDGs post COVID-19 are provided including the following: (1) sustainability action plan considering COVID-19 implications: refining sustainability goals and targets and developing measurement framework; (2) making the most of sustainability transition opportunities in the wake of COVID-19; (3) innovative solutions for economic resilience toward sustainability post COVID-19; (4) in-depth analysis of the COVID-19 long-term effects on social sustainability; and (5) expanding quantitative research to harmonize the COVID-19-related sustainability research.

Another research study was undertaken by Wang and Huang (2021) to comprehensively examine articles linked to the influence of COVID-19 on sustainability in the Web of Science database using bibliometric tools. The findings revealed that the research was dominated by developed countries although the epidemic offered more severe obstacles to achieving sustainable development in underdeveloped nations than industrialized countries. Developed nations were dedicated to investigating the long-term education viability but developing countries had focused on economic viability during the pandemic. The cluster analysis also revealed that the COVID-19 pandemic had negative consequences for 17 SDGs, but the pandemic might potentially provide opportunities for 14 SDGs. Finally, the researchers offered appropriate ideas for attaining SDGs in the post-epidemic period.

Many research studies have investigated COVID-19 emergency management and epidemic prevention and control techniques. Mathematical and statistical models have been utilized for modeling disease outbreaks to help in disaster planning and response decision-making. In addition, these models have been critical in designing risk management methods that reduce the spread of disease outbreaks and other undesirable consequences, such as shortages of vital resources and economic downturns. System dynamics simulation and modeling approach is a holistic method for mapping interactions in complex systems, including nonlinearities, feedback loops, and delays (Edaibat et al. 2017). For instance, Beigi (2020) developed a system dynamic model to study the correlation between the SDGs and COVID-19 preventative strategies. This novel qualitative approach reinvigorated debates about the future of SDGs amid the crisis. Besides, it provided a powerful mental representation for decision-makers to find leverage points that aid in preventing long-term disruptive effects of the health crisis on people, environment, and economy. The research highlighted the necessity of conducting more quantitative and qualitative scientific research to quantify the importance of attaining the SDG objectives in individual nations based on continuing lessons learned from the health crisis.

Numerous research studies have revealed a strong correlation between COVID-19 spread and unsustainable conditions with excessive air pollution (Coccia 2020). Jia et al. (2022) focused on the harm COVID-19 poses to human health and society. The authors applied system dynamics to create a prevention and control model that incorporated material supply, public opinion dissemination, public awareness, scientific and technological research, staggering work shifts, and warning effect (of law/policy). To uncover relationships between subsystems and investigate the major elements impacting social benefit, causal loop analysis was employed. Different scenarios were also dynamically simulated to find the best combination modes. The following were the key findings: (1) the low supervision mode would cause a lag in material delivery and a superimposed effect on social benefit, and (2) the strong supervision mode offered many benefits, including the ability to minimize online public opinion propagation, concealment, false declaration rates, increase government credibility, and social benefit. However, in the middle and late stages, the effect of intensive monitoring would progressively decrease, necessitating correction. The findings could serve as a theoretical foundation for bettering epidemic prevention and control strategies.

Sy et al. (2020) developed a system dynamic modeling method that encompassed connections, feedbacks, and delays. The effectiveness of different measures in reducing infection and the consequent economic burden was studied and appraised. According to preliminary modeling, the most successful methods focused on preventing viral exposure in the first place; expanding healthcare capacity merely delayed the ultimate system collapse since its efficacy presupposed that individual became sick first. The research suggested the application of system dynamics to get a better knowledge of other elements of the system, such as the optimum method to handle hospital operations in the event of a pandemic. Furthermore, it recommended the utilization of other modeling approaches (e.g., optimization modeling) for resource allocation, particularly when vital medical supplies and commodities were scarce. It may also be used to choose among viable policy options and operationalize their execution based on several competing stakeholder goals.

There have been few research studies on the application of system dynamics to the COVID-19 epidemic. To address this research gap, this research proposes developing a web-based simulation model to analyze the impact of the COVID-19 pandemic on the SDGs in Egypt using system dynamics. This research study attempts to address four main objectives: (1) describe the application of a systems dynamics approach to the COVID-19 pandemic, (2) predict the trend of COVID-19 spread in the short and long term in Egypt, (3) assess the ramifications of COVID-19 on the SDGs given the uncertainty about its characteristics, and (4) evaluate the effectiveness of government response strategies on the pandemic's outcome and the achievement of SDGs. This model is intended to provide decision-makers with an evidence-based judgment that assists the government in formulating effective and equitable policies and actions in the pandemic era. Deeper analysis and evaluation frameworks are critical for supporting decision-making for risk management strategies and transmission control measures that take into account the interconnections across participating sectors.

## Materials and methods

The Corona Virus Impact Assessment Model (COVIAM) using system dynamics approach is used in contexts where standard analysis is difficult given the wide range of available data and/or relationships in place. In particular, it would be specifically helpful in systems, which are highly influenced by soft variables connected to human behavior.

The systemic approach, which closely follows the systems thinking and system dynamics methodology prescriptions, has allowed for a simple yet very effective representation of such context, with an identification of those parameters that, in a “domino effect”, influence the behavior of the whole interconnected system. The proposed system dynamics approach can provide decision-makers with a useful tool to understand and evaluate some of the expectable risks triggering critical events. In the last few years, the system dynamics approach has been used to manage public health issues and many other domains. Besides, this simulation approach is capable of inherently taking into account randomness and interdependency, which both characterize the behavior of real-life environments. The idea behind the system dynamics approach is that, if “a system structure defines its behavior” (Sterman 2001), then by being accurate in analyzing and determining the interrelationships among various parts of the system, it could be possible to define accurately the structure of the problem under study and this would ultimately bring an increased understanding of the dynamics of the system. Thus, the system dynamics approach replicates dynamic systems in real life with the power to “look into the future” and to understand the impact on multiple key metrics. Additionally, simulation allows the user to capture the specific variability of multiple processes and ultimately provides results, which are orders of magnitude more accurate than deterministic analysis. This research used a system dynamics approach to simulate the nonlinear behavior of COVID-19 impacts on public health and the related SDGs.

**Fig. 1:**
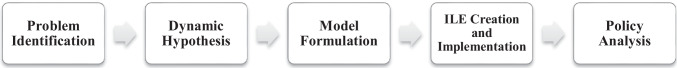
System dynamics model process

Five-step methodology will be set up and implement to model complex system’s behaviors in critical events and to achieve the challenging task endowed by the COVIAM model as shown in Fig. 1. A detailed description of the steps of the process is presented in the following subsection.

### Research setting and hypotheses

The proposed simulation model can simulate the impact of the COVID-19 pandemic on the SDGs in the short and long term in Egypt. In this research, the model simulation is conducted over 35 years extending from 2015 to 2050. The next paragraphs will go through the model in detail as well as their hypothesis.

#### Problem identification

**Fig. 2:**
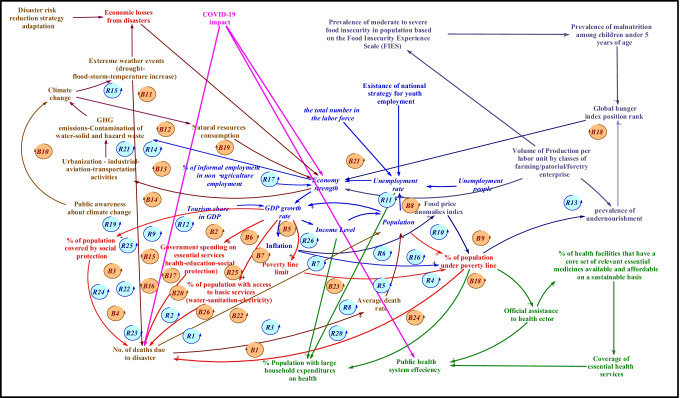
Causal loop structure of the proposed model

To simulate the impacts of COVID-19 on sustainability goals and provide a more reliable picture of their effects, all essential variables that affect the system are considered as depicted in Fig. 2 which represents the conceptual framework of the COVIAM model. The framework is considered all essential variables related to sustainable goals that are chosen to investigate their effects on the sustainability of public health, which are SDG 1 (no poverty), SDG 2 (zero hunger), SDG 8 (decent work and economic growth), and SDG 13 (climate action).

#### Dynamic hypothesis

Examining the impact of the COVID-19 pandemic on the selected goals of sustainable development public health in Egypt, in the long run, requires examining the effect of the major variables on the assessment. This is done by using a tool that is capable of visualizing relationships of variables and feedback effects of the system. The structure of the system dynamics model is portrayed by a causal loop diagram, which is formulated by VENSIM software as shown in Fig. 2. The developed causal loop diagram model comprises fifty-two causal loops in total; twenty-six of them are positive causal loops, and the other twenty-six are negative. The system’s ultimate behavior is determined by the interactions between distinct loops that capture the impact of COVID-19 on the SDGs.

Feedback loops B1 and B2 depict the relationship between the number of deaths due to disasters and the proportion of the population under the poverty line where the number of dead people increases as a result of disasters increases average mortality rate, this leads to a decrease in population numbers. This results in a decrease in the percentage of citizens who are below the poverty line. On the other hand, when the percentage of citizens who are below the poverty line declined, it leads to a decrease in the number of deaths as a result of disasters from the previous number. This relationship, as a natural result of research findings developed by Johnson (2017), people who lie below the poverty line are more likely to lack access to a key resource needed for living, more vulnerable in the face of disasters, and are more likely to suffer more serious consequences during impact, from property damage to homelessness to physical and financial impacts. By referring to feedback loop B3, it can be observed that raising the number of people that die as a result of disasters reduces the population, which lowers the GDP growth rate. This in turn leads to strengthening social protection policies and ensuring efficiency in social spending. Consequently, the number of deaths due to disasters will be decreased due to increasing the percentage of the population covered by social protection. Feedback loop B4 is the same causal loop relationships of loop B3, but the difference in effects of the average annual death rate on population where the increase in deaths from different risks leads to an increase in the average annual death rate. By referring to feedback loop B5, it can be observed that increasing the number of dead people leads to a decrease in population numbers. This results in decreasing the unemployment rate, strengthening the economy, and rising GDP growth rate. As a result, the proportion of the population with access to basic services will be increased. This results in decreasing the number of deaths from the risk of lack of basic services necessary for livelihoods. Feedback loop B6 is the same causal loop relationships of loop B5, but the difference in effects of government spending on essential services instead of the proportion of beneficiaries of the population to access basic services. Feedback loop B7 is the same causal loop relationships of loop B5, but the difference in the negative effect of the average annual death rate on population.

Feedback loops R1 and R2 show that increasing the number of deaths due to disaster helps in reducing the population, which leads to a decrease in the GDP growth rate where there is a direct positive correlation between population growth and economic growth (Sibe et al. 2016). This is reflected in reducing the government spending on basic services such as water, sanitation, and electricity leading to a decrease in the proportion of beneficiaries of the population to access to these basic services. This results in increasing the number of deaths from the risk of lack of basic services necessary for livelihoods. Some of the causal loop relationships in loop R3 are the same as in loop R2; the difference is that the increase in deaths from different risks leads to an increase in the average annual death rate, which is reflected in a decrease in population growth. This has negative effects on economic growth. Consequently, if economic growth is low, then, the government spending on basic services is low. Feedback loop R4 shows that there is a negative relationship between deaths number and total population, where increasing number of deaths decreases the total number of population. This in turn leads to a decrease in the GPD growth rate. Thus, it will increase the value of inflation. Inflation indicates that prices have increased. Inflation affects the purchasing power of money, which reduces consumption and, as a result, the GDP growth rate (Mukoka 2018). Consequently, the number of deaths due to disasters will be decreased due to increasing the percentage of the population covered by social protection. Thus, when inflation increases, it leads to an increase in the proportion of the population below the poverty line, which increases the chance of an increase in deaths as a result of disasters. Feedback loop R5 is the same causal loop relationships of loop R4, but the difference in effects of the average annual death rate on population where the increase in deaths from different risks leads to an increase in the average annual death rate.

Feedback loop R6 is the same causal loop relationships of loop R2, but the difference in effects of the average annual death rate on population where the increase in deaths from different risks leads to an increase in the average annual death rate. It can be shown from feedback loop R7 that increasing the number of individuals who die as a result of disasters reduces the population and lowering the GDP growth rate. This results in decreasing the minimum level of income (Ravallion 1992). This in turn leads to raising the total number of people under the poverty line. Accordingly, this is an indicator of an increase in the poor class and an increase in the number of people exposed to peril as a result of disasters. Feedback loop R8 is the same causal loop relationships of loop R7, but the difference in effects of the average annual death rate on population where the increase in deaths from different risks leads to an increase in the average annual death rate. Feedback loop R9 has the same causal loop relationships of loop B5, but the difference lies in the negative effect of increasing GDP growth rate on the proportion of the population covered by social protection, which leads to an increase in deaths from different risks leading to an increase in the average annual death rate. Feedback loop R10 is the same causal loop relationships of loop R4, but the difference in the positive effects of inflation on rising food price anomalies index. This leads to increasing the population under the poverty line. As a result, an increase in deaths from different risks will be found. Feedback loop R11 is the same causal loop relationships of loop R8, but the difference in the positive effect of decreasing the unemployment rate on economic strength.

### Sample and data

To simulate the long-term impacts of COVID-19 crises on SDGs, all essential variables that affect the system are considered. The data of SDGs related to Egypt that were used in this research were extracted from different sources such as World Bank, Egyptian Central Agency for Public Mobilization and Statistics (CAPMAS), and UN (see Table 1).

**Table 1:** Descriptions of model variables

No	SDG	Variable	Unit	Initial value (2015)
1	SDG 1: No poverty	Proportion of the population below the poverty line	%	27.8
2		Proportion of population with access to basic services	%	Improved water source = 98
			%	Electricity = 100
			%	Improved sanitation facilities = 93
3		Proportion of government spending on essential services	%	5.59
4		Proportion of population covered by social protection (TAKAFOL Program)	Person	2,173,274
5		Proportion of population covered by social protection (KARAMA Program)	Person	1,239,732
6	SDG 2: Zero hunger	Prevalence of undernourishment	%	4.6
7		Indicator of food price anomalies (from inflation)	%	10.99
8		Prevalence of moderate or severe food insecurity in the population	%	27.76
9		Volume of production per labor unit by classes of farming/pastoral/forestry enterprise size	$US	NA
10		Prevalence of malnutrition among children under 5 years of age	%	9.48
11	SDG 8: Decent work and economic growth	Inflation rate	%	10.37
12		Annual GDP per capita growth rate	%	5.43
13		Tourism share in GDP	%	8.7
14		Unemployment rate	%	13.05
15		Proportion of informal employment in non-agriculture employment	%	30.94
16	SDG 13: Climate action	Number of deaths due to disasters	person	40
17		Greenhouse gas (GHG) emissions per year due to energy, imports, and fossil fuel exports	mton	264.9
18		Level of adaptation of disaster risk reduction strategy among local government units, which include planning, financing, and implementing the strategy	Dmnl	0.2
19		Level of public awareness about climate change and environmental issues that is improved by government activities such as planning, financing, and implementing	Dmnl	0.3

Source: World Bank, Egyptian Central Agency for Public Mobilization and Statistics (CAPMAS), UN

### Measures of variables

The initial values of variables used in the model have been collected for base year (2015). The change rates of different variables according to the Egyptian current path and the 2030 vision are considered. Detailed descriptions of the model variables are included in Table 1.

### Model and data analysis procedure

**Fig. 3:**
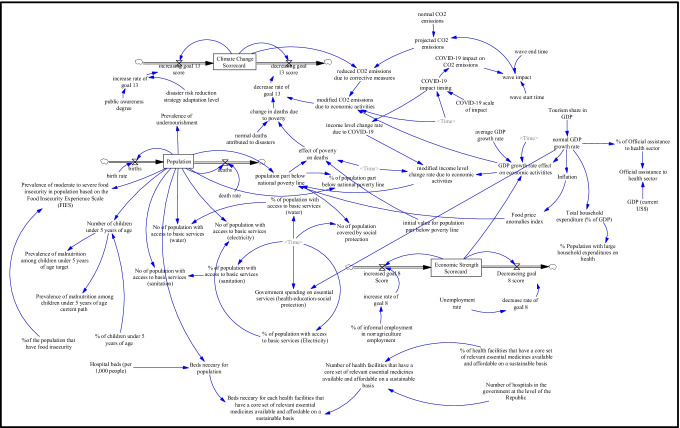
A stock-flow diagram for assessing the impact of the COVID-19 pandemic on the SDGs

Based on the causal loop diagram, all the key variables that affect the behavior of the SDGs under the impact of the COVID-19 pandemic are identified. The conceptual causal loop diagram is converted to a quantitative model to simulate the running of the model. To this end, the causal loop diagram is converted into a stock-flow diagram using VENSIM software. The main components of the stock-flow diagrams that represent the variables under consideration and their equations are stock, flow, converter, and connector. A stock variable is a noun that refers to something that builds up through time, such as population, climate change scorecards, and economic strength scorecards. A flow is an activity that fills and drains a stock, such as birth reparents represent the filling and deaths represent the draining for the population. The direction of positive flow is indicated by the unfilled arrow head on the flow pipe. A flow’s value can be positive or negative. The converter represents the other numbers used to define external inputs to the model, such as the birth rate and death rate, which are utilized to create the model's equations. The connector links model elements together. Clouds are used to show that a flow's drain is outside the model's bounds. The proposed stock-flow diagram depicts dynamic structures between COVID-19 and the SDGs, which are represented by various icons and arrows. These indicators and connectors are clearly defined and linked to differential equation structures that were used to forecast the crisis's effects. Figure 3 depicts a stock-flow diagram of the developed model.

### Interactive learning environment creation

A web-based interactive learning environment (ILE) is created to help decision-makers analyze the interdependencies among public health activities, which are affected by unpredictable catastrophic events, and study the impacts of possible intervention countermeasures or prevention policies. The ILE can be easily tailored and transformed into a Decision Support System (DSS) effectively usable in a specific environment, both by disaster preparedness analysts and by personnel acting in disaster management control rooms. The interface has the objectives of enabling the users to exploit the functionalities of the model without the need of being expert modelers and to organize the model’s inputs and outputs for further analyses performed with spreadsheet technologies.

The selected software to create the ILE is Forio Epicenter. It was launched in 2014 as a useful platform that allows users to upload simulation models in different languages and create interactive user interfaces for education, forecasting, or predictive analytics without any required knowledge about system dynamics or modeling background. The created interfaces are HTML 5-based interfaces that can be either private, shared with a specific number of authenticated users, or even provided to the general public and include templates for the ease of creation. They can be accessed on any computer, mobile phone, or tablet.

**Fig. 4:**
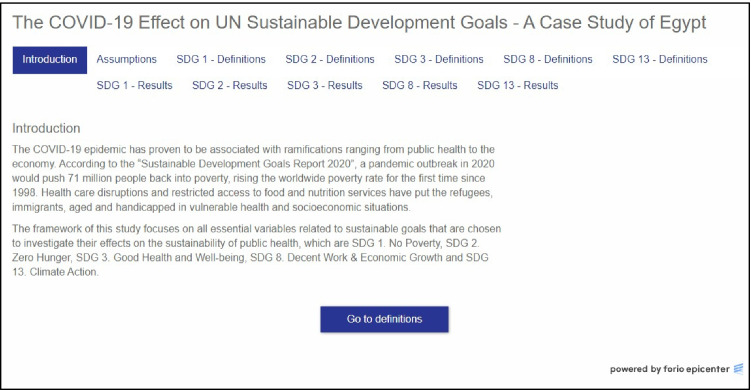
COVIAM model–based ILE with system dynamics

The chosen project type is the “Run Comparison” project. This type of project suits the time-based models and provides the opportunity for the user to insert different initial assumptions and run a comparison to answer “what if” questions. The created interface in this research includes four parts shown in the project tabs: (1) introduction and assumptions applied on the studied SDGs, (2) brief explanation of the studied indicators for non-expert users, (3) visualization of the results of the simulation in the form of graphs, and (4) a textual representation and discussion of the results (see Fig. 4).

## Results and discussion

The model simulation is conducted over 35 years extending from 2015 to 2050. This helps use the Egyptian current path and the 2030 vision to predict Egypt’s performance throughout the studied period. The most important simulated results of the most effective indicators in the experts’ opinions are represented from the built Forio Epicenter user interface.

**Fig. 5:**
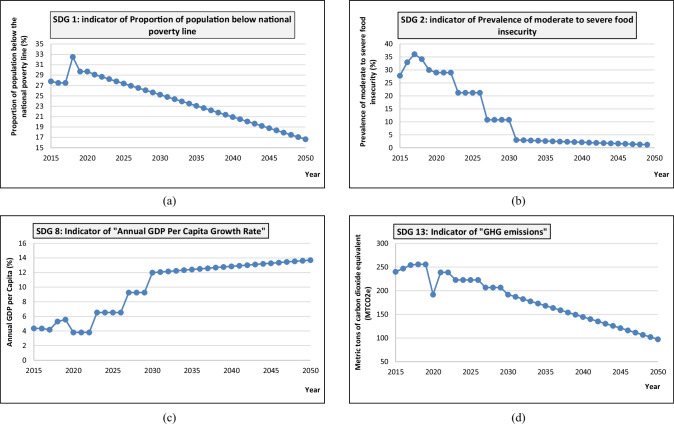
Expected COVID-19 impact in Egypt with respect to **a** SDG1, **b** SDG2, **c** SDG8, and **d** SDG13

To study Egypt’s performance toward the “no poverty” SDG, the indicator 1.2.1: Proportion of population living below the national poverty line is studied (Fig. 5). Figure 5 shows that the percentage of the population under the poverty line was 27.5 in 2015 and stayed stable to the year 2017 and increased significantly to be 32.5% in 2018. It should be noted that average poverty line per capita in Egypt rose to LE 735 ($44.4) per month for the year 2017/2018, compared to LE 482 ($29.1) per month in 2015. The main reason for the increase in poverty rates by 4.7%, during the period between 2016 and 2018, is the implementation of the economic reform program during the same period, which required a cost to society and the Egyptian state. After 2018, Egypt’s poverty rate fell to 29.7% in 2020. With the continuation of the state's efforts to improve the individual's livelihood and achieve the SDGs, the poverty line rate is expected to decline until it reaches around 25% by 2030 and it will continue to decline until it reaches approximately 16% by 2050.

The second targeted SDG is “zero hunger” which can be clearly expressed by the indicator 2.1.2: Prevalence of moderate or severe food insecurity in the population, based on the Food Insecurity Experience Scale (FIES), shown in Fig. 5, showed a stable response to the COVID-19 impact on the Egyptian economy. Despite the overall worldwide increase in food insecurity, results show that Egypt maintained a constant percentage of around 29% from the start of the pandemic up till now. According to this significant governmental effort to follow its vision of 2030 where Egypt vision is a “first step toward inclusive development,” to ensure that future societal transitions are both inclusive and sustainable, Egypt can achieve a decreasing percentage of food insecurity and can reach 3% food insecurity in 2030 and this decrease will continue until it reaches full sufficiency by 2050.

From 2015 to 2019, Egypt managed to increase its GDP growth rate from 4.37 to 5.56%. Due to the COVID-19 circumstances, Egypt’s economy was significantly affected. This is represented by a sudden contraction in the GDP growth rate that occurred in 2020 and 2021 to a value of 3.8%. Following Egypt’s vision of 2030, this negative impact is expected to be stabilized and a higher projected 2030 value of 11.99% can be achieved by an average annual increase of 0.91%. According to global studies and estimates, the globe will seem very different in 2050 than it does now. Egypt's economy has the potential to outperform others. It outperforms a large number of countries around the world. Egyptian GDP could reach $4.333 trillion by 2050 if proper policies are implemented from the current path ($394.8 billion) (Hussain 2017). For that, according to the simulation model, the GDP growth rate is expected to increase yearly to reach 13.71% by 2050 as shown in Fig. 5.

To track Egypt’s performance related to the “climate action” SDG, indicator 13.2.2: a total GHG emission per year was studied as shown in Fig. 5. Starting at a value of 240 Mt CO2-equivalents in 2015, GHG emissions show a slight increase followed by a sudden temporary decrease to a value of 192 Mt CO2-equivalents in 2020 due to the lockdown impact on economic activity. After the lockdown was over in 2021, the GHG emissions showed a significant increase compared to 2020 by reaching a value of 239 Mt CO2-equivalents. This finding is in line with Ramadan and Ramadan’s study results (2021), in which their geographical model revealed that in the year 2021, the environmental scenario rated best in highly exposed areas to COVID-19 outbreaks. Following Egypt’s vision 2030 and by committing to the Paris Agreement regarding reducing global warming, it is expected that GHG emission will tend to decrease in the following years until a value of 192 Mt CO2-equivalents is achieved again in 2030. According to Climate change mitigation initiatives, it calls for a 50–80% reduction in emissions by 2050 compared to 1990 levels that reached around 20,000 Mt CO2-equivalents (World Bank Group 2016). Owing to Egypt has a share of 0.75% of global emissions (Crippa et al. 2019), GHG emissions are expected to decrease to around 97 Mt CO2-equivalents by 2050. These cutbacks are the result of the Egyptian government's efforts to preserve growth while dealing with the COVID-19 shock. In recent years, the majority of government responses to the COVID-19 epidemic have focused on risk mitigation techniques and activating governmental efforts to raise public awareness about climate change and environmental concerns.

## Conclusion

COVID-19, a novel coronavirus disease, poses a severe threat to reaching the SDGs. In order to address this issue, a system dynamic model is developed to examine the pandemic’s impact on four SDGs in Egypt. SDG 1 (no poverty), SDG 2 (zero hunger), SDG 8 (decent work and economic growth), and SDG 13 (climate action) are among the addressed targets. The model simulation takes place for 35 years, from 2015 to 2050. This enables the use of Egypt’s current trajectory and the 2030 goal to forecast Egypt’s performance over the study period. The main findings of this research are summarized in the following subsections.

In 2015, the percentage of the population living in poverty was 27.5%, which remained steady until 2017 before rising to 32.5% in 2018. The average poverty line per capita increased to LE. 735 ($44.4) per month in 2017/2018, up from LE. 482 ($29.1) per month in 2015. The implementation of the economic reform program over the same period is the main reason for the 4.7% increase in poverty rates between 2016 and 2018. The poverty rate declined to 29.7% in 2020 and it is predicted to fall until it reaches around 25% by 2030. It will continue to decline until it reaches around 16% by 2050 if the efforts to enhance individual livelihoods and achieve the SDGs are maintained. Despite a global increase in food insecurity, Egypt maintained a consistent ratio of roughly 29% from the start of the epidemic until now. Egypt can achieve a decreasing percentage of food insecurity, reaching 3% in 2030, according to the significant governmental efforts to follow its vision of 2030. This percentage will continue to decrease until it reaches full sufficiency by 2050.

Egypt’s economy was heavily impacted by COVID-19, as seen by a sharp drop in GDP growth rate to 3.8% in 2020 and 2021. Following Egypt’s 2030 vision, this negative impact is expected to be stabilized, with an average annual growth of 0.91% achieving a higher projected 2030 value of 11.99%. If the right policies are adopted, GDP might reach $4.333 trillion by 2050, up from $399.8 billion now. According to the simulation model, the GDP growth rate will rise every year until it reaches 13.71% in 2050.

The total GHG emission per year was evaluated to track Egypt’s performance in relation to the “climate action” SDG. Starting at 240 Mt CO2-equivalents in 2015, GHG emissions exhibit a small increase before dropping to 192 Mt CO2-equivalents in 2020 due to the economic activity lockout. Following the end of the lockout in 2021, GHG emissions increased significantly, achieving 239 Mt CO2-equivalents. Following Egypt’s Vision 2030 and its commitment to the Paris Agreement on climate change, it is predicted that GHG emissions will decline in the coming years, returning to a value of 192 Mt CO2-equivalents in 2030. GHG emissions are predicted to fall to roughly 97 Mt CO2-equivalents by 2050.

The developed model aimed at providing decision-makers with an evidence-based judgment that will aid the government in establishing effective policies and measures. Furthermore, it re-energized discussions about achieving SDGs in the midst of the crisis and served as a useful tool for decision-makers in identifying leverage points to minimize the crisis’ long-term detrimental effects on the economy, people, and environment. The proposed methodology could be generalized to other countries, undertaking the world commitment to the 2030 Agenda for Sustainable Development. To achieve the desired goals of sustainable development in the face of crises, actors should get involved in all relevant activities to identify obstacles, determine priorities, align actions, and mobilize resources in order to create the necessary flexibility. They shall develop medium- and long-term plans to monitor and counteract monopolistic practices, as well as to strengthen regulatory bodies’ roles. Finally, they have to enhance policy integration to take advantage of synergies across economic, social, and environmental objectives.

In terms of research restrictions, one of the problems that stymies attempts to anticipate progress toward the SDGs is the data vacuum problem surrounding crises and their quality. The proposed methodology also has limitation in that it does not provide specific outputs for the effects of COVID-19 crises on SDGs, instead displaying anticipated future trends, orientations, and relative magnitudes of influence.

## Data Availability

Not applicable.
